# Social Injustice in the Neoliberal Pandemic Era for Homeless Persons With Mental Illness: A Qualitative Inquiry From India

**DOI:** 10.3389/fpsyt.2021.635715

**Published:** 2021-06-17

**Authors:** Prama Bhattacharya, Gunjan Chandak Khemka, Laboni Roy, Sarbani Das Roy

**Affiliations:** ^1^Department of Humanities and Social Sciences, Indian Institute of Technology Kanpur, Kanpur, India; ^2^Iswar Sankalpa, Kolkata, India

**Keywords:** COVID-19, social injustice, homeless persons with mental illness, constructivist grounded theory analysis, India

## Abstract

The Corona Virus Disease 2019 (COVID-19) pandemic has presented an unprecedented challenge globally. It is much bigger than a bio-medical concern now with the multitudes of socio-economic, socio-political, socio-cultural, and psycho-social impact, which are likely to outlast the pandemic itself by far and long. The pandemic and the resulting challenges across societies highlighted the existing social injustices in a neoliberal world for historically marginalized populations like homeless persons with mental illness (HPMI). The nationwide lockdown in India to resist the spread of the virus posed a unique challenge to this vulnerable population. The present study thus attempts to understand the experience of HPMI during the COVID-19 induced lockdown through the theoretical framework of social justice vis-à-vis injustice. Semi-structured interviews have been conducted on seven HPMI rehabilitated in the community through an NGO situated in Kolkata, India. Seven stakeholders have also been interviewed to understand their experience in providing services to the HPMI during the COVID-19 induced lockdown. Analyses of the narratives have been done using initial coding, focused coding and axial coding through the process of constant comparison of constructivist grounded theory (CGT) methodology. Critical insights from the study bring out experiences of HPMI during COVID-19 as a victim of structural violence, highlighting their exclusion and victimization due to the existing marginalized status, living closer to the edge as a consequence of the lockdown, lack of awareness of the gravity of the pandemic situation. The experiences of the stakeholders, on the other hand, pointed out the role of community members and social workers in partially mitigating the challenges. This study indicates that to mitigate the aftermaths, stakeholders, including community members, need to work together for rebuilding and enhancing the strength and resilience of the marginalized populations like HPMI, who are historically victims of social injustice in the neoliberal pandemic era.

## Introduction

It took a pandemic for the global community to discuss mental health, global inequalities, social injustices, healthcare disparities, and many such issues that we were comfortable not talking about. The Corona Virus Disease 2019 (COVID-19) is much bigger than a bio-medical concern now. When the virus started spreading earlier this year, creeping through the Wuhan province border in China, even the most experienced epidemiologists across the world did not presume the toll to be this severe. Though the global message has been that of “*we are all in this together,”* the reality is far from this emotion. Beyond the epidemiological jargon, the socio-economic, socio-political, socio-cultural, and psycho-social impact of the pandemic has affected different nations differently across cultures, communities, and sections of their respective societies. Politicians often referred to COVID-19 as a “great equalizer,” focusing on the sentiment that the virus has affected everyone regardless of their age, socio-economic status, gender, or ethnicity (1). However, one cannot turn a blind eye toward the differential impact of the virus on historically marginalized groups like migrant laborers, slum dwellers, homeless individuals, internally displaced persons, or the likes.

International and national crises like pandemics, wars, or tsunamis often highlight inequalities that may have been overlooked or hidden during the absence of the crisis. Times of crisis exacerbate these disparities, making them a sore point for stakeholders. COVID-19 and the resulting challenges across societies have showcased inequities and discrimination experienced by most vulnerable populations.

It is not a novel situation for marginalized communities in a lower-middle-income country (LMIC) like India to live on the edge with their life, security, physical and mental health at risk consistently. However, the COVID-19 pandemic made this experience more intense for them with a sudden withdrawal of the most accessible resources. Migrant laborers forced into reverse migration (2), homeless people evicted from the shacks they call *home* *(**3**)*, older adults (4), and children forced into loneliness and restriction, women being subjected to increased domestic violence (5), and the list continues.

### The Homeless Persons With Mental Illness in India During COVID-19

An invisible burden to society, the homeless persons are often conveniently overlooked in a country's census, making it easier for stakeholders to ignore their responsibilities and accountability. Earlier pandemics like SARS and Influenza documented that the homeless population pose a unique threat to themselves as well as public health. Leung et al. (6) identified that the rate of spread of infection and mortality was much higher in this population. However, the percentage of diagnosis and treatment was much lower than in the general population. The poor living condition leading to poor immunity makes them more vulnerable to get affected by any form of illness, including COVID-19. Because of their fleeting nature, it becomes a challenge for the stakeholders to quarantine them once affected, and thus they might also become an accessory to the spread of disease (7).

In an LMIC like India, the homelessness scenario is much more critical. It houses a significant percentage of the world's homeless population. The Census of India in 2011 reported 1.77 million homeless in the country that accounts for the 0.15 percentage of her total population (8), which many researchers consider being under-reporting “due to the lacunae in enumeration” [(9), p. 11]. Action Aid, in a survey conducted in 2003, estimated that the homeless population in India is 78 million (9). There are also a high number of mentally ill individuals living across the streets of India, forming a significant chunk of the “homeless” crowd. There are at least 1.77 million homeless as per the Census of India, 2011 (8), the estimated number of homeless people suffering a severe mental illness (SMI) becomes on the verge of half a million.

### Social Justice in the Neoliberal Pandemic Era for Homeless Persons With Mental Illness

Homeless persons with mental illness (HPMI) is one of the most vulnerable population in the global health platform. With the advent of the unprecedented pandemic situation, their vulnerabilities have increased manifold. Not only they are more prone to being affected by COVID-19, but that also makes them a threat to public health as well. Their unique marginalized position intensifies their suffering due to structurally induced social injustices. Levy and Sidel (10) conceptualized social injustice as “the denial or violation of human rights (economic, socio-cultural, political, or civil rights) of specific populations or groups in society based on the erroneous perception of their inferiority by those with more power or influence” (p. 3). As proposed by (10), Social Justice, on the other hand, is based on principles of distributive justice, “the equitable societal distribution of valued goods and necessary burdens. Social justice applies the concept of distributive justice to the assets, privileges, and advantages that are present in society” [as cited in (10) p. 5].

The causes of public ill-health are often a complex interplay of myriads of factors, many of which are because of social injustice, which includes poverty, inadequate education, inadequate healthcare services, and inaccessibility to many essential resources. The neo-liberal push toward individualized responsibility for illness and recovery, disguised as freedom and agency, has been exacerbating the already existing social injustice in the health sector as the HPMI end up struggling for services, amenities, and welfare irrespective of the pandemic. In the context of the present study, neoliberalism is conceptualized as a socio-cultural pattern including a political-economic agenda that emphasizes limited government and deregulation of markets, and a “cultural ideology emphasizing freedom over other liberal values (e.g., equality)” [(11), p. 4]. In contrast to the neo-liberal trends, the social justice framework of public health advocates the concept that governments are *established* for the benefit of their citizens, and they *must* provide for and protect their welfare, including upholding their human rights. Social justice is the primary philosophy based on which the understanding of public health has been built (12), at the core of which lies the need to minimize preventable illness, injury, and premature death through equal rights to access healthcare resources. Health equity, a concept closely related to social justice, as identified by Levy and Sidel (10), “is the absence of systematic inequalities in health, or in the major social determinants of health, among groups at different levels in the social hierarchy in terms of wealth, power, and/or prestige” (p. 5).

Being “deficient,” “dependent,” “disabled,” or “dysfunctional” is disdainful in a world which objectifies people as “products” and “resources.” Having a mental illness (MI) in a world marked with economic and social inequalities earns one a label of being an “irremediable loser”. The neoliberal world has been increasingly identifying people with MI as “consumers” of mental healthcare services. Further, obsessed with medicalization, it continues to perceive “mental illness” as a problem within the individual, overlooking the social, cultural, and economic dynamics) (13). Such individualized responsibility for illness and recovery, disguised as freedom and agency, leads to alienation and loneliness, as people with MI end up fighting alone for services, amenities, and welfare. In a developing country like India, the struggle to avail the barely enough resources to survive for those living with the double burden of mental illness along with homelessness is superimposed upon the pre-existing battle for a healthier physical, social, and psychological and economic existence. The pandemic situation posed by COVID-19 has made this marginalized position yet more prominent. Pandemic or not, the HMPIs are ostracized by the hegemony of the “disability lens” imposed upon them by the neoliberal state and the society. The pandemic has not only refocused the attention toward the existing social injustice and inequalities in a neoliberal world but intensified them as well. It thus becomes critically relevant to understand through the theoretical framework of social justice vis-à-vis social injustice how the unjust neoliberal context might have shaped the experiences of the HPMIs during the COVID-19 pandemic.

### The Context of the Present Study

#### Iswar Sankalpa

Iswar Sankalpa, an NGO established in 2007, is based in Kolkata, West Bengal, India. The organization was formed to reduce the existing gap between the large number of HPMI in Kolkata and the limited medical and psycho-social resources available. It has been working “to ensure the dignity and holistic well-being of persons with psycho-social disabilities, particularly to those from underprivileged parts of society, in a humane manner, and in addition empower them in attaining their rights” [(14), p. 1]. Like in most other parts of the country, Kolkata also provides psychiatric care primarily through clinical services available in hospitals. Following the overarching neoliberal trends in the healthcare system, the onus of accessing health care is placed solely on the patients and their caregivers. As a direct consequence, HPMI is often rendered without treatment because of the absence of a primary caregiver, and with little or no insight, they are often unable to fend for themselves. Before Iswar Sankalpa, there was no coordinated effort to address this consistently increasing gap.

#### Naya Daur

Of all the programmes run by the NGO (for example, male and female shelter-houses, assisted living for persons with psycho-social disabilities in a sustainable community, Urban Mental Health Programmes in collaboration with the Kolkata Municipal Corporation), “Naya Daur” (the New Run) or the outreach programme targeting community-based rehabilitation is the earliest. It started the same year the NGO was founded. The core philosophy of this programme is to mobilize the existing community resources in taking an active in taking responsibility for the rehabilitation of HPMI through community integration. With minimal intervention and optimal use of community resources, the aim is to ensure dignity, freedom and self-determination among the rehabilitated persons.

The programme was tailor-made in the purview of the sociocultural context of the city of Kolkata. The inquisitive neighborhood feeling that lies core to the spirit of Kolkata has been one of the critical factors that give Iswar Sankalpa the vision for its outreach programme. The community at Kolkata has always been interested in finding out what is happening to the person next to it, and that makes the community engage with everything around, from football to tea-stall “adda” (gatherings), to politics, to taking care of the disheveled “pagol” (mad person) in the locality. An HPMI is often identified as a “nuisance” or “menace” by the community. Thus, instead of letting the person remain on the street while being treated, community members almost always ask service providers to “take them away” from that locality. However, the programme has helped, to a large extent, in sensitizing the community and developing a massive pool of proxy-caregivers on the streets of Kolkata by slowly and gradually involving the community.

### Epistemological Stance

As mentioned earlier, the present study aims to represent the unheard voices and experiences of the HPMI, suffering and surviving the COVID-19 pandemic. To collect data and present numbers, we often choose to *un-hear* the voices of those whom we claim to represent. The importance of giving precedence to the voice of the re-searched has become immensely significant in the newer/alternative paradigms as they advocate for a researcher- participant relationship, the use of vantage points of the researcher toward enculturing action, and the power of research to promote “social justice, community, diversity, civic discourse, and caring” [(15), p. 278].

The theoretical standpoint as that of social constructionism for the present study thus ensured that the voice of the research participants would be heard in the way they want themselves to be heard, not the way the researcher wants them to speak (16). It also provides the scope to explore their experiences within the same sociocultural context that has constructed their experiences of being a victim to structurally induced violence beyond the pandemic as well.

### The Present Study

As highlighted earlier, in India, the COVID-19 pandemic and the consequent nationwide lockdown played havoc on historically marginalized populations, with the HPMI being no exception. Academicians have been offering a theoretical understanding of the challenges HPMIs might experience because of the pandemic and the lockdown as a victim of structurally induced ostracizations (3, 7, 17). However, there is a lack of representation of their lived-experience as a victim of structural violence during the pandemic. With the help of the “Naya Daur” programme team of Iswar Sankalpa, the present study aims to bring forward the voices of these homeless persons as they continue suffering and surviving the pandemic and the consequent lockdown. The following broad research objectives have been conceived toward this end:

1) Understanding the experiences of HPMI during the COVID-19 pandemic induced lockdown as a victim of structural violence.
2) Understanding the role of stakeholders in providing services to the HPMI during the COVID-19 pandemic induced lockdown.

## Methodology

Ethnography lets the researcher understand the worldview of the people from their vantage point (18). Ethnography, thus, appeared to be a suitable choice as a qualitative methodology in addressing the objectives of the research that are centered on bringing forward the lived experience of HPMI during the COVID-19 pandemic induced lockdown.

### Fieldwork

In ethnographic research, the fieldwork forms the crux of the journey the researcher has set forth. The Naya Daur programme comprises seven zones spread all over the city of Kolkata. Along with the respective social worker and counselor assigned to each zone, the first author started visiting the outreach fields of Iswar Sankalpa to interview the HPMIs and observe their daily routine within their natural setting during September 2020. Most of the individuals stayed in the open on the roadside pavements, at bus stops, under the flyover and bridges, while some managed to arrange a shack for themselves. The NGO attempted to provide them with essential resources like medicines, clothes, food materials, and masks (as the pandemic mandated). While a few of these individuals had started earning their livelihood by working as daily wage earners before the pandemic struck, many of them were solely dependent on the services provided to them by the NGO and the community members. It would be discussed later how they experienced the disruption in this service consequent to the pandemic.

### Participants

At present, the Naya Daur programme caters to 120 HPMIs across these zones. However, it was logistically not possible to interview with all of them, mainly because many of them would still be actively symptomatic. The psychiatric diagnoses were made by the treating psychiatrists appointed by the NGO when the individuals were included into the programme. Thus, after a mindful deliberation with the respective project coordinator, counselors, and social workers, seven participants (Table 1) were selected who might provide an insight into the research objective under exploration. Only those individuals were included in the study who had no active psychotic symptoms (in remission) and declared fit for the study by the treating psychiatrist. However, information concerning eventual psychiatric or physical comorbidities, as well as details of ongoing treatments were not available.

**Table 1:** Preliminary Information about HPMIs.

**S.No**.	**Participant**	**Age/Gender**	**Psychiatric Diagnosis**
1	Ramesh	49/M	Psychosis NOS
2	Shekhar	43/M	Schizophrenia
3	Abhishek	31/M	Schizophrenia
4	Babul	40/M	Psychosis NOS
5	Paul	42/M	Bipolar Affective Disorder
6	Bela	47/F	Schizophrenia
7	Anwara	44/F	Bipolar Affective Disorder

Simultaneously, proxy caregivers from a few of the zones were interviewed along with the social workers and counselors to understand their roles in providing services during the COVID-19 pandemic (Table 2). All the participants consented to the interviews. Pseudonyms have been used for those participants who are HPMI.

**Table 2:** Preliminary Information about Stakeholders.

**S. No**.	**Participants**	**Service Provided**
1	Shankar	Proxy-caregiver, community member
2	Ranjit	Proxy-caregiver, community member
3	Banani	Proxy-caregiver, community member
4	Ms. S	Counselor
5	Mr. D	Social worker
6	Ms. M	Naya Daur Project Coordinator
7	Mr. SH	Social worker

### Ethics

The principle of informed consent and complete confidentiality was applied. Ethical approval from the Internal Ethics Committee of Iswar Sankalpa was taken. No physical test was done.

### Data Collection

A large chunk of the field visits was spent observing the social lives of the HPMIs in their natural setting as they continued with their daily chores, interacted with fellow street dwellers, community members, the social workers, and counselors, or sit idle oblivion to the world around them. The semi-structured interviews were interspersed in between these observations and interactions as and when the counselor or social worker informed me about the readiness of the participants for that.

The semi-structured interviews were developed, keeping in mind the research objectives. For the HPMI participants, the interviews initiated with general probes related to their personal history (How long have you been staying here? How did you end up being on the streets?), their daily routine and activities as a way of understanding life on the streets and break the ice. Through open-ended questions, the interview then probed around their experiences of the lockdown, starting with whether they were mindful of why the lockdown happened if they observed any changes around them during and following that phase and if that has affected their lives.

Discussions then were initiated on whether they experience any new challenges at present, their coping strategies, the role of the social workers and community members during this time. They were also asked if they had heard of COVID 19, what they knew about it, and how did they learn about that information?

The semi-structured interviews with the social workers, counselors and community members were centered around the objective of understanding their role in mitigating the challenges the HPMIs might have experienced during the COVID-19 pandemic induced lockdown. The stakeholders also provided significant insights into the challenges they experienced and their observations on the experiences of the HPMIs.

## Constructivist Grounded Theory Analysis of Data

An ethnographer undergoes an array of rich experience before, during and after the fieldwork, seeing data everywhere, gathering everything and thereby having the risk of piling up “mountains of unconnected data,” which do not say much. Thus, as an analytical method, the strategies of Constructivist Grounded Theory can help the ethnographer arrange, compile, and gain insights into the collected data in a more holistic way, increasing the “analytic incisiveness” (19).

As identified and explained by (20–23), the processes of coding, constant comparison, theoretical sampling and theoretical saturation of CGT method help the researcher to “unravel the nuances of human experiences or worldviews within their relational, cultural and socio-political contexts” [(24) p. 402].

Following the transcribing, the data required to be coded first. Charmaz [(22), p. 43] conceptualized coding as “categorizing segments of data with a short name that simultaneously summarizes and accounts for each piece of data.” A typical CGT analysis would include two types of coding, initial, focused and sometimes axial coding. Charmaz [(22) p. 46] defined initial coding as “naming each word, line, or segment of the data.” Focused coding decides about “which initial codes make the most analytic sense to categorize your data incisively and completely” [(22) p. 58].

**Figure 1:**
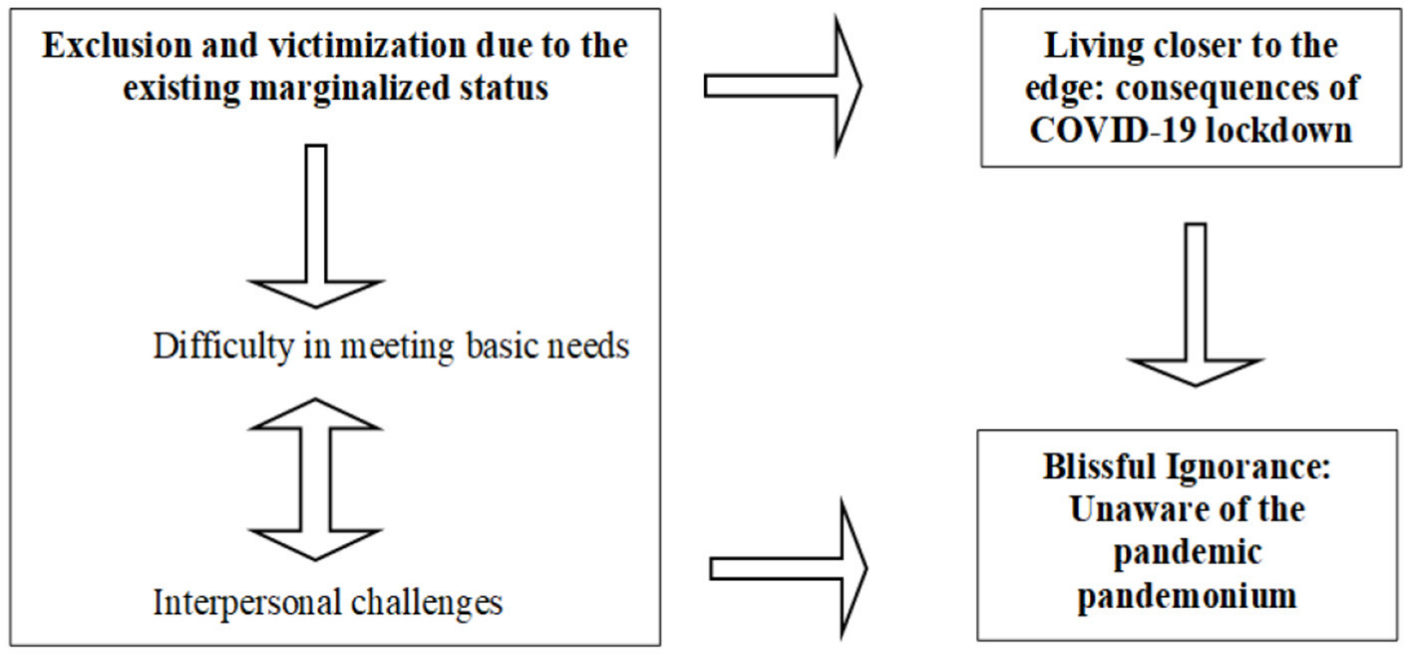
Schema to understand the experiences of HPMI during COVID-19 as a victim of structural violence.

**Figure 2:**
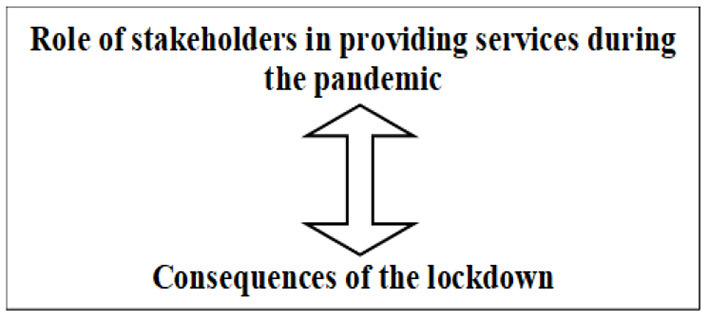
Schema to understand the role of stakeholders in partially mitigating the challenges experienced by HPMI during COVID-19.

Finally, the focused codes that best represent the data are considered (22). The other focused codes that are grouped under one category are then termed as their sub-categories. For example, while the focused code “*Exclusion and victimization due to the existing marginalized status”* becomes a category, the other focused codes like “difficulty in meeting basic needs” and “interpersonal challenges” become its sub-categories. The CGT analysis helped in developing the final categories that represented the data to explore the present research objectives. The following categories (Figure 1) were developed to understand the experiences of HPMI during COVID-19 as a victim of structural violence: Exclusion and victimization due to the existing marginalized status (Difficulty in meeting basic needs, Interpersonal challenges), Living closer to the edge: consequences of COVID-19 lockdown and Blissful Ignorance: Unaware of the pandemic pandemonium. The following categories (Figure 2) were developed through CGT analysis to understand the role of stakeholders in partially mitigating the challenges experienced by HPMI during COVID-19: Role of stakeholders in providing services during the pandemic and Consequences of the lockdown.

### Categories to Understand the Experiences of HPMI During COVID-19 as a Victim of Structural Violence (Figure 1)

#### Exclusion and Victimization Due to the Existing Marginalized Status

Feeling of helplessness and dehumanization on experiencing the inability to take care of oneself due to resourcelessness that was exacerbated during the COVID-19 lockdown due to

a) Difficulty in meeting basic needs
b) Interpersonal challenges.

#### Living Closer to the Edge: Consequences of COVID-19 Lockdown

Experiencing further deterioration of their marginalized status following the COVID-19 lockdown phase as a victim of structural violence that intensified due to the pandemic.

#### Blissful Ignorance: Unaware of the Pandemic Pandemonium

Being unaware of the gravity of the pandemic situation either due to inadequate access to information or being unable to maintain the protocol because of being marginally situated.

### Categories to Understand the Role of Stakeholders in Partially Mitigating the Challenges Experienced by HPMI During COVID-19

#### Role of Stakeholders in Providing Services During the Pandemic

The proactive role of community members and social workers during and following the pandemic induced lockdown in providing basic amenities to HPMI that were inaccessible to them because of their marginalized status.

#### Consequences of the Lockdown

Experiencing extensive difficulty in providing service due to the unprecedented challenges posed on their way due to the lockdown phase of the COVID-19 pandemic.

## Findings

### Experiences of HPMI During COVID-19 as a Victim of Structural Violence

The following section explores the experiences of the HPMI during COVID-19 as a victim of structural violence.

### Exclusion and Victimization Due to the Existing Marginalized Status

The HPMI expressed feeling helpless and dehumanized by experiencing the inability to take care of oneself due to resourcelessness exacerbated during the COVID-19 lockdown.

#### Difficulty in Meeting Basic Needs

While resourcelessness was nothing new for them, with the help of the NGO and concerned community members, many of these individuals, who still stayed on the streets, had become able to cater to their essential requirements. For them, the lockdown took away the very basics of survival—and health concerns remained alien to many as they struggled to access food and water. Babul, who worked at a factory before the lockdown, became dependent on others (NGO and Community) as the lockdown started. He recollected,

These people (social workers) were also not coming when the lockdown started. For the first one or two days, there was no food. Then, people started coming and distributing food. We (along with other homeless street dwellers) used to collect and store those. One time, the food that I had stored became stale. I ate that without knowing that it was stale. I got severe diarrhea.

The prescribed medicines that he was on also got discontinued as the social workers could not visit him.

The medicines that I take daily was also not there since they (the social workers) were coming. It used to feel strange inside my head [mathay kirokom onnyorokom hoto]. (Babul, 40/M, Diagnosed with Psychosis NOS)

A similar experience was shared by Ramesh, who too worked at a factory, “All these shops were closed… The factory in which I work was also closed. It was weird. There was no work to do. My medicines got over” (Ramesh, 49/M, Diagnosed with Psychosis NOS).

Abhishek, an HPMI who also happened to have an injured leg, generally earned his livelihood by begging at the roadside. When the lockdown started, he remembered, suddenly there was no one around to give him a penny, nor a shop was open to buy food from, “Initially, when the lockdown began, for the first two days, I had no food. I survived on the tube-well water. There was not a single shop open for me to go and buy food” (Abhishek, 31/M, diagnosed with Schizophrenia).

Shekhar, who worked as a daily-wage earner at a local market, also found it challenging to arrange his food,

It was difficult to arrange food during that lockdown. I used to go from one place to the other in search of food. People were coming with food relief after some time. I used to find them out and get some food (Shekhar, 43/M, Diagnosed with Schizophrenia).

#### Interpersonal Challenges

Social isolation and coping with it have been one of the novel situations experienced globally. However, while most individuals could share their distress with their family members, friends or neighbors through one-to-one, digital or social media connections, neither these facilities nor the social support were available to the HPMI. Most of them live alone on the streets in the company of the community, without a family. The lockdown resulted in a sudden withdrawal of this community connection. All the hustle-bustle of the streets, the fellow homeless neighbor, the inquisitive shopkeepers, the concerned community members, the regularly visiting social worker, everything “vanished” without any prior notice. As Abhishek recollected, “Suddenly all the shops were closed. There was not a single car or bus on the street. There was no one around. I could not talk to anyone for days, sitting here all day long…it used to feel so strange” (Abhishek, 31/M, diagnosed with Schizophrenia).

Bela remembered how they were not allowed on the roads by the authorities. It was indeed another new experience because, for nearly a decade, she has known the streets to be her home.

I remember that they (the social workers) were not coming. All the shops closed down. All of a sudden, there was no one around. It was all so silent. The trains stopped running. We were not allowed on the streets. Police used to come and chase us away if we were sitting on the pavements. They arrested some people. (Bela, 47/F, Diagnosed with Schizophrenia)

### Living Closer to the Edge: Consequences of COVID-19 Lockdown

As discussed earlier, the HPMI are already one of the most marginalized sections globally. They survive with the most minimal resources in all segments of their life. However, the nationwide lockdown that followed the onset of the pandemic had a rather debilitating impact on their already marginalized status. They experienced a further deterioration of their marginalized situation following the COVID-19 lockdown phase as a victim of structural violence that intensified due to the pandemic. Loss of job, inaccessibility to food and medicines, loss of community support was rampant as the lockdown had impacted the socio-economic stability across societal strata. For example, the factories or roadside tea stalls they worked in were closed. Community members like the local shopkeepers who provided them with some support were out of business. The social workers were not allowed to visit them when the lockdown started. As a consequence, the HPMI were living closer to the edge than ever before. Babul shared his experience of surviving the lockdown and the months following that

I lost the job I had. The factory in which I worked has not opened yet. I have been begging for the last seven months sitting on the roadside. But hardly anyone gives a penny. We survive on tea and bread. When they (social workers) come, they get me food. (Babul, 40/M, Diagnosed with Psychosis NOS).

### Blissful Ignorance: Unaware of the Pandemic Pandemonium

The narratives of the HPMI showed that most of them were unaware of the gravity of the pandemic situation either due to inadequate access to information,

It is a disease. It is like tiny insects. I do not know how it is spreading. You can get that information from the television. But no one can tell you why it is happening. God controls all these things in this world (Abhishek, 31/M, diagnosed with Schizophrenia)

or were unable to maintain the protocol because of being marginally situated,

I have not heard much about this. They (the social workers) are asking to put on a mask on the face. They got me one. But mine got stolen along with every other thing I had. They ask me to stay clean, but what can I do to keep clean when I sleep on the pavement and sit on the roadside all day long! (Paul, 42/M, Diagnosed with Bipolar Affective Disorder)

### Role of Stakeholders in Partially Mitigating the Challenges Experienced by HPMI During COVID-19

It was evident that the HPMI experienced plenty of challenges posed in their everyday life due to the pandemic and the resulting lockdown. However, various stakeholders like the social workers, proxy-caregivers (community members) played a significant role in partially mitigating the difficulties experienced by the HPMI.

### Role of Community Members and Social Workers in Providing Essential Resources

The community members and social workers took up a proactive role during and following COVID-19 lockdown in providing basic amenities to HPMI that were otherwise inaccessible to them because of their marginalized status. Shankar, a roadside tea-stall owner, had been a proxy caregiver to Shekhar for 2 years. Even after Shekhar got a job for himself after he embarked upon his journey toward recovery, Shankar continued being social support for Shekhar. Thus, when the lockdown started and his factory closed down, Shekhar turned to Shankar for support despite whose own shop closed down for business.

I looked after Shekhar when he was ill, two years back. Now he was doing well, working, and earning money. But when the lockdown started, the factory closed down, and he had nowhere to go. I made him stay with me. But my tea stall was also closed. I hardly had money to fend for myself. So, I started selling fruits and make some money. It was challenging to make ends meet. But we managed. The social workers were not able to visit him. His medicines got over. I managed to buy some from the local pharmacy. The social worker instructed me over the phone to do so. (Shankar, roadside tea-stall owner)

Even when she had difficulty making ends meet, community members like Banani continued supporting fellow homeless neighbors like Anwara.

I stay by the railway track where Anwara (an HPMI) stays. She had severe diarrhea some time back. I looked after her then. I worked as a house-help but lost the job when the lockdown started. My husband, a daily wage earner, also lost the job. Now I pick rags and sell them. When the lockdown began, I also fed Anwara with whatever little I could. The didi (social worker) who gave her food was not coming. I had to do something for her. You cannot see someone starve to death in front of you. Whatever I boiled, some rice and potato, we shared with her as well. (Banani, homeless community member)

Similarly, the social workers went out of their way, risking their well-being to provide the essential support to the HPMI they were responsible for. Even amidst the lockdown, they continued visiting them as much as they could afford.

The lockdown began so suddenly; I had no clue how to manage things with my homeless clients for the first week. They do not have mobile phones. We were not allowed outside our homes. But then, after a week, I decided I need to go and see them. I bought some dry food and mineral water bottles and went to see them. It was a risk, but I had to go. (Mr SH, social worker, Iswar Sankalpa)

The NGO sought help from the community through social media platforms and received some positive responses.

When the lockdown started, we reached out to the community through social media urging them to provide food to any HMI individuals around them if they can. We got some positive responses to that. We have also been receiving some donations from the community. (Ms. M, Project Coordinator, Iswar Sankalpa)

Despite all their efforts to support the HPMI during the pandemic, the difficulties of providing services to them increased manifold. Apart from existing challenges like lack of resources, awareness and support at a systemic level, they experienced some newer difficulties as they attempted to continue their services irrespective of the lockdown and pandemic. Losing some of the HPMI they were providing services to, have been one such challenge.

Since this is a very transient, fleeting population, we lost some clients during the lockdown's initial three-week phase. Maybe they went in search of food and could not trace their way back. We have not been able to relocate them yet. (Ms. S, counselor, Iswar Sankalpa)

## Discussion

There is empirical evidence that living in unequal societies with some people being much worse off, economically and socially, tends to produce deprivations in the absolute quality of life that people enjoy.

- Amartya Sen, 2017 while delivering a public lecture in Ohio State

### Social Injustice Toward the HPMI in the Neoliberal Pandemic Era

Even though social injustice is a global phenomenon, it becomes evident in LMICs like India, where there is an extreme income disparity. Oxfam, India in 2020 highlighted that with India's top 1% population holding 42.5% of her total wealth, the gap widened further during the pandemic crisis (25). Rising inequality compromises access to all form of social, economic, education and health opportunities. Despite the mainstream acceptance and awareness of the socio-economic impact of this vast inequality and consequently injustices, meaningful remedies continue to remain elusive. As already identified in the introduction, the neoliberal world's perception of mental illness as individualized responsibility has already, without considering the social, political and economic context, already intensifies the suffering of the individuals with mental illness. The marginalized position of the HPMI further magnifies this experience of social injustice.

Globally, homelessness focuses on increasing social and public health concern, even in countries with adequate infrastructure. A significant proportion of the homeless population suffers from mental health issues, making it more difficult for them to take care of their own needs without support. As a consequence, the creeks of social injustices and inequality grow deeper and deeper. A victim of structurally induced social injustice, marginalized populations like the HPMI are often denied access to their basic human needs being met.

The situation became worse than ever with the onset of the pandemic. Irrespective of their mental health condition, COVID-19 pushed the HPMI further toward the edge. To control the spread of the virus, the Government decided on a nationwide lockdown. Little did they consider the impact it would have on those who have been struggling against the domination of the neoliberal state that perceives them as consumers of the services. With the lockdown, as they lost their job, their minimal social support, and in some cases two times meals(“Exclusion and victimization due to the existing marginalized status”), the HPMI could not care less about how an invisible virus would affect them while they are already victimized by the hidden pandemic that is homelessness (“Blissful Ignorance: Unaware of the Pandemic Pandemonium”). Intensifying their experiences of being dehumanized further, in the name of social isolation, homeless individuals were forcefully evacuated from the streets to shelter-houses which had neither the adequate means nor resources to maintain the protocol for COVID-19. Unavailability of pertinent information regarding safety protocol made the pandemic situation yet more obscure for the HPMI as they struggled to arrange the day's meal (“Living closer to the edge: Consequences of COVID-19 lockdown”).

### The Role of Stakeholders in Bridging the Gap

India has in her core embedded the very philosophy of serving the sufferer. The concept of “*Seva”* (serving) runs through the veins of her sociocultural rootedness, irrespective of the diverse religions, language or culture. Institutions like The Ramakrishna Mission or The Missionaries of Charity, amongst others, have been translating this age-old philosophy into action by delivering their unconditional services to the destitute. As the neoliberal society and its stakeholders turn a blind eye toward the social injustice that it causes to marginalized population like HPMI, the role of the NGO sector becomes crucial in bridging the gaps and providing services to such socially alienated people.

Of many overlooked problem areas in developing countries, mental health is undoubtedly one. Patel and Thara (26) identified the vast responsibility that lies with the NGO sector to bridge this gap. While India on paper has since then taken various initiatives (signing the UNCRPD in 2006, Mental Healthcare Act of 2017) to meet the challenges of the burden that the increased number of untreated mentally ill citizens pose on her, implemented actions are still missing. Thus, the role of the NGOs remains as relevant as it was two decades back. Though (26) pointed out their importance, they further highlighted the lack of the number of NGOs who are committed to the cause of serving the mentally ill population. Nevertheless, the dedication of those working relentlessly in this area despite all odds (including lack of funding) is never fully acknowledged.

While working in the field of mental illness can be complex and challenging, it is yet even more challenging to serve those who have mental illness and are homeless at the same time. The homeless population are, in the first place, very fleeting. Furthermore, identifying the one who has a mental illness, though not difficult, a diagnosis remains crucial and must involve a medical practitioner. The psychiatrist-patient ratio in India is highly inadequate, and thus, finding a trained professional for the NGO sector often becomes difficult. Moreover, because of the lack of caregiver, treatment and serving the HMI involves a medico-legal context. Simultaneously, unlike many other marginalized populations, a lot of taboo and stigma exists around mental illness even today. Thus, rehabilitation of the HMI, even when possible, is unwelcomed at various sectors of society. With so many hurdles to tackle, structured initiatives to serve the HMI in the NGO sector are considerably much lower than what is needed.

The social workers engaged with the Naya Daur programme of the Iswar Sankalpa have been doing their bit. The inclusive culture of Kolkata and the decades of work that this NGO has been doing of creating a community that would cater for its own paid off during the pandemic. Even when the proxy caregivers could not afford a “full belly meal” for their own selves during the lockdown and even now, they continue to provide for the HPMI. Such community involvement is the portrayal of the age-old philosophy that lies at the core of the Indian culture (“Role of community members and social workers in providing essential resources”). It gives us hope at the grimmest of these hours when the neoliberal state washes their hands off their responsibility to the last person(s) on the streets, and the policies fail their purposes of serving those for whom they were meant to be.

## Conclusion

Marginalized populations are quickly forgotten. It is only because of the COVID-19 pandemic that we are discussing at length how they are victimized by structural forces, income inequalities and social injustice, all wrapped together by neoliberal hegemony.

Not all homeless people end up having a mental illness; neither all individuals who have mental illness reach the point of homelessness. Nevertheless, the lack of access to resources makes it more difficult for them to exit the vicious cycle of homelessness- severe mental illness. Myriads of other factors like unemployment, migration, power processes within a patriarchal society, gender-based violence, and the like play havoc on the experiences of the HPMI as they live their life of homelessness coupled with SMI. However, it is beyond the scope of the present article to explore all these factors keeping in mind the present research objective. It might be considered a future research implication where the role of these factors might be explored in the HPMI population in the context of the pandemic.

Hopefully, with time, the bio-medical impact of the pandemic would be over as the medical community invent the vaccine. Nevertheless, the socio-economic, socio-cultural and socio-political, and psycho-social implications that it has been causing to the society would outlast the pandemic by far and long. Furthermore, like all calamities, the brunt would be most intensely experienced by the marginalized sections. To mitigate the aftermaths, as Pandey et al. (2) explicated, stakeholders need to work together for rebuilding and enhancing the strength and resilience of the survivors of structurally induced violence. Characteristically, the neoliberal state would silence the voice of the survivors of the structural violence, underplaying their suffering, medicalizing their experiences. For that, more than ever, acknowledgment of social inequality and injustice at the systemic level is needed because the “*ostrich effect*” would not help, not anymore.

## Data Availability Statement

The raw data supporting the conclusions of this article will be made available by the authors, without undue reservation.

## Ethics Statement

The studies involving human participants were reviewed and approved by Internal Ethics Committee, Iswar Sankalpa. The patients/participants provided their written informed consent to participate in this study.

## Author Contributions

PB has been responsible for data collection, data curation, data analysis, and the first draft of the manuscript. GK, LR, and SR have been responsible for the supervision, revision, and editing of the draft. All the authors conceptualized the study. The final draft of the manuscript has been read and approved by all the authors.

## Conflict of Interest

The authors declare that the research was conducted in the absence of any commercial or financial relationships that could be construed as a potential conflict of interest.

## References

[1] Kantamneni N. The impact of the COVID-19 pandemic on marginalized populations in the United States: a research agenda. J Vocat Behav. (2020) 119:103439. 10.1016/j.jvb.2020.103439 32390658 PMC7205696

[2] Pandey R Kukreja S Priya KR. COVID19. Mental Health Migrant Work. (2020) 55:16.

[3] Banerjee D Bhattacharya P. The hidden vulnerability of homelessness in the COVID-19 pandemic: perspectives from India. Int J Soc Psychiatry. (2020) 10.1177/0020764020922890. [Epub ahead of print]. 32408789 PMC8191169

[4] D'cruz M Banerjee D. 'An invisible human rights crisis': the marginalization of older adults during the COVID-19 pandemic-an advocacy review. Psychiatry Res. (2020) 292:113369. 10.1016/j.psychres.2020.113369 32795754 PMC7397988

[5] Nair VS Banerjee D. “The Cries Behind the Closed Rooms”: Domestic Violence Against Women During COVID-19, A Crisis Call. Bangalore: NIMHANS.

[6] Leung CS Ho MM Kiss A Gundlapalli AV Hwang SW. Homelessness and the response to emerging infectious disease outbreaks: lessons from SARS. J Urban Health. (2008) 85:402–10. 10.1007/s11524-008-9270-2 18347991 PMC2329752

[7] Kar SK Arafat SY Marthoenis M Kabir R. Homeless mentally ill people and COVID-19 pandemic: the two-way sword for LMICs. Asian J Psychiatr. (2020) 51:102067. 10.1016/j.ajp.2020.102067 32305034 PMC7194975

[8] Goel K Chowdhary R. Living homeless in urban India: state and societal responses. In: Faces of Homelessness in the Asia Pacific. Routledge (2017). p. 47–63.

[9] Sattar S. Homelessness in India. Shelter-Hudco Publication (2014). p. 9–15.

[10] Levy BS Sidel VW editors. Social Injustice and Public Health. NewYork, NY: Oxford University Press (2013).

[11] Adams G Estrada-Villalta S Sullivan D Markus HR. The psychology of neoliberalism and the neoliberalism of psychology. J Social Issues. (2019) 75:189–216. 10.1111/josi.12305

[12] Foege WH. Public health: moving from debt to legacy. Am J Public Health. (1987) 77:1276–8. 10.2105/AJPH.77.10.1276 3631359 PMC1647124

[13] Esposito L Perez FM. Neoliberalism and the commodification of mental health. Hum Soc. (2014) 38:414–42. 10.1177/0160597614544958

[14] Iswar Sankalpa Annual Report 2016-2017,. (2017). p. 1. Available online at: https://isankalpa.org/document/annual-reports/

[15] Lincoln YS. Emerging criteria for quality in qualitative and interpretive research. Qual Inquiry. (1995) 1:275–89. 10.1177/107780049500100301

[16] Sampson EE. Identity politics: challenges to psychology's understanding. Am Psychol. (1993) 48:1219. 10.1037/0003-066X.48.12.1219

[17] Gowda GS Chithra NK Moirangthem S Kumar CN Math SB. Homeless persons with mental illness and COVID pandemic: collective efforts from India. Asian J Psychiatr. (2020) 54:102268. 10.1016/j.ajp.2020.102268 32622032 PMC7313514

[18] Hammersley M Atkinson P. Ethnography: Principles in Practice. London: Routledge (2007).

[19] Charmaz K Mitchell RG. Grounded theory in ethnography. In: Atkinson P Coffey A Delamont S Lofland J Lofland HL editors. Handbook of Ethnography. California: Sage (2001). pp. 160–74.

[20] Charmaz K. The body, identity, and self: adapting to impairment. Sociol Q. (1995) 34:657–80.

[21] Charmaz K. Constructivist and objectivist grounded theory. In” Denzin NK Lincoln SY editors. Handbook of Qualitative Research. California: Sage (2000). pp. 509–35.

[22] Charmaz K. Constructing Grounded Theory: A Practical Guide Through Qualitative Research. London: Sage (2006).

[23] Charmaz K. Constructing Grounded Theory. London: Sage (2014).

[24] Priya KR. Using constructivist grounded theory methodology: studying suffering and healing as a case example. In: Bryant AJ Charmaz K editors. The SAGE Handbook of Current Developments in Grounded Theory. London: SAGE Reference (2019). 392–412.

[25] Lawson M. Coronavirus and Inequality. (2020). Retrieved from: https://www.oxfamindia.org/blog/coronavirus-and-inequality (accessed November 30, 2020).

[26] Patel V Thara R. Introduction: the role of NGOs in mental health care. In: Patel V Thara R editors. Meeting the Mental Health Needs of Developing Countries: NGO Innovations in India. Sage Publications India (2003). p. 1–19.

